# A Study on the Properties of Composite Modified Mortar with Styrene–Butadiene Rubber Latex and Silica Fume

**DOI:** 10.3390/polym16050697

**Published:** 2024-03-04

**Authors:** Renwei Yan, Laifa Wang, Yongjun Ni, Shuowen Zhang, Zhenqing He, Bowen Guan

**Affiliations:** 1 Qinghai Guoluo Highway Engineering Construction Co., Ltd., Xining 810021, China; rwyanqhjk@163.com (R.Y.); yongjuniguoluo@163.com (Y.N.); 2 Qinghai Provincial Traffic Control Construction Engineering Group Co., Ltd., Xining 810001, China; laifawang2001@163.com; 3 School of Materials Science and Engineering, Chang’an University, Xi’an 710061, China; 2021131062@chd.edu.cn (S.Z.); 2022131068@chd.edu.cn (Z.H.)

**Keywords:** cement mortar, styrene–butadiene rubber latex, silica fume, road performance, microstructure, synergistic effect

## Abstract

To solve the problem of the poor abrasion resistance of concrete pavement surface mortar, this study substituted cement with equal amounts of styrene–butadiene rubber (SBR) latex and silica fume (SF) to investigate the effects of organic/inorganic material composite modification on the fluidity, drying shrinkage, mechanical properties, and abrasion resistance of cement mortar. Also in this study, the microstructure, product, and pore structure characteristics of the composite modified cement mortar were investigated using scanning electron microscope (SEM), X-Ray diffraction (XRD), Fourier-transform infrared spectroscopy (FT-IR), and the Brunauer–Emmett–Teller (BET) method. This research found that the sole substitution of SF negatively impacted the mortar’s fluidity and drying shrinkage yet enhanced its mechanical strength and abrasion resistance; the incorporation of SBR latex improved fluidity, reduced shrinkage, and increased flexural strength but adversely affected the compressive strength of the mortar. Additionally, the enhancement of the mortar’s abrasion resistance with SBR latex was significantly greater than that with SF. When SBR latex and SF were used together as substitutes, the latex struggled to offset the negative impact of SF on mortar fluidity but effectively reduced shrinkage; SF compensated for the detrimental effect of the latex on compressive strength. Moreover, the primary role in enhancing the mortar’s abrasion resistance was played by the latex. Microscopic tests showed that SBR latex and SF could increase the content of calcium silicate hydrate (C-S-H) gel, inhibit the formation of ettringite (AFt) and reduce carbonation, refine the pore size of cement mortar, and effectively improve the microstructure of mortar.

## 1. Introduction

With the rapid development of highway traffic and the exponential growth in traffic volume, the share of concrete pavement in the transportation sector is gradually increasing. In comparison to asphalt pavement, concrete pavement exhibits excellent fatigue resistance and superior durability. However, poor skid resistance and susceptibility to wear hinder the wider application of concrete pavement [1]. Generally, as cement mortar controls the area of contact between the pavement and the tires, ensuring good friction, it is imperative to design cement mortar with excellent wear resistance [2].

Researchers commonly enhance the wear resistance of concrete pavement through means such as improving aggregate performance [3,4,5], surface treatments [6,7], adding fibers [8,9,10], and incorporating supplementary cementitious materials [11,12]. Among existing methods, the addition of SF is a relatively practical and environmentally friendly approach. Yon [13] achieved optimal results in wear resistance for self-compacting mortar by substituting 20% silica fume (SF) and 10% slag. Jain [14] observed a reduction of 11.05% in concrete wear at the substitution level of 10% SF and 20% iron powder. Onuaguluchi [15] significantly improved the wear resistance of rubberized cement mortar by replacing 10% of the volume with SF. However, the enhancement of concrete or mortar wear resistance with SF is based on relatively high blending ratios (≥10%), and an excessive proportion of SF in the cementitious material system can lead to severe shrinkage issues [16,17,18], adversely affecting the road performance of concrete.

Apart from the aforementioned methods, the use of polymer emulsions to enhance the performance of cement mortar has become a common practice. Studies indicate that polymer emulsions can increase mortar’s flexural strength, bond strength, and flexibility [19,20]. Additionally, polymers demonstrate favorable performance in enhancing mortar durability, including resistance to freeze–thaw cycles, chloride ion penetration, carbonation, and chemical erosion [21]. Styrene–butadiene rubber (SBR) latex, as one of the most commonly used polymers in cementitious materials [22], exhibits outstanding performance in certain aspects. Chen et al. [23] found that both SBR latex and carboxylated SBR latex have a positive effect on the wear resistance and skid resistance of road cement mortar. Moodi et al. [24] discovered that an appropriate amount of SBR latex not only improves the wear resistance of concrete but also enhances shrinkage and freeze–thaw resistance. However, extensive research [19,25], including the use of polymers like SBR latex, indicates adverse effects on the compressive strength of cement-based materials. Therefore, the combined use of polymers with mineral admixtures, fibers, or nanomaterials has become a new and promising research direction.

This study employs SBR latex and SF to individually and jointly replace cement in cement mortar, investigating the changes in flowability, shrinkage, mechanical properties, and wear resistance of cement mortar after composite modification with organic/inorganic materials. Simultaneously, this study utilizes testing methods such as scanning electron microscope (SEM), X-Ray diffraction (XRD), Fourier-transform infrared spectroscopy (FT-IR) and the Brunauer–Emmett–Teller (BET) method to investigate the microstructure, products, and pore structure characteristics of composite-modified cement mortar, aiming to understand the synergistic effects of SBR latex and SF in cement mortar.

## 2. Materials and Methods

### 2.1. Materials

The cementitious materials used in this study include Portland cement of the type P.O 42.5 R, produced by Beijing JinYu Co., Ltd., Beijing, China. SBR latex produced by Shenzhen Yoshida Chemical Co., Ltd., Shenzhen, China. and the first level of SF in China. The chemical composition of the cement and SF are shown in Table 1. The SBR latex has a solid content of 50%, pH value of 7.8~10, viscosity of 35~150 mPa·s, and a minimum film-forming temperature of 15~21 °C. Additionally, organic silicone-based defoamer was used, with 20~40 mesh quartz sand as the fine aggregate. Organic silicone defoamer and quartz sand were supplied by Jinan JingBo Biotechnology Co., Ltd, Jinan, China. The materials above were all mixed with tap water.

### 2.2. Mix Design

SBR latex and SF were used to replace cement of an equal mass at proportions of 5%, 10%, and 15%. The water-to-binder ratio was fixed at 0.4, and the binder-to-sand ratio was 1:2. The amount of defoamer used was 0.6% of the total amount of cementitious materials. The experimental water consisted of all the water in the styrene butadiene latex except the solids. Additionally, four sets of experiments with combined dosages of SBR latex and SF were designed. The baseline specimen was named CM; specimens with SBR latex and SF used individually and specimens with the combined substitution were named Ln, Sn, and LnSm, respectively, where n and m denote the substitution rates. Specimens for determining mortar fluidity, drying shrinkage, mechanical properties, and abrasion resistance were prepared with the same mix proportions. All of the specific proportions are shown in Table 2.

### 2.3. Testing Methods

#### 2.3.1. Fluidity

The flowability of cement mortar was carried out according to the “Testing Methods of Cement and Concrete for Highway Engineering” (JTG 3420-2020) [26].

#### 2.3.2. Drying Shrinkage

The drying shrinkage of cement mortar was tested according to the “Testing Methods of Cement and Concrete for Highway Engineering” (JTG 3420-2020) [26], with specimen dimensions of 25 mm × 25 mm × 280 mm. The specimens were demolded after 24 h of curing, and the lengths of the mortars were measured at 1, 7, 14, 28, 56, 90, 120, and 150 days after demolding to calculate the drying shrinkage rate using the following Equation (1):

(1) $$D_{t}=\frac{L_{0}-L_{t}}{L_{0}}$$

where $D_{t}$ is the drying shrinkage rate when the time is *t*; $L_{0}$ is the sample’s initial length (mm); $L_{t}$ is the sample’s length when the time is *t* (mm). The drying shrinkage of three mortar samples was measured, and the average value was obtained.

#### 2.3.3. Mechanical Properties

The mechanical properties of cement mortar were tested according to the “Testing Methods of Cement and Concrete for Highway Engineering” (JTG 3420-2020) [26], with specimen dimensions of 40 mm × 40 mm × 160 mm. A flexural strength test was first conducted with a loading rate of 0.5 mm/min and a span of 150 mm. The broken specimens were then used for compressive strength testing.

#### 2.3.4. Abrasion Resistance

The abrasion resistance of mortar was conducted in accordance with the “Testing Methods of Cement and Concrete for Highway Engineering” (JTG 3420-2020) [26], with specimen dimensions of 150 mm × 150 mm × 30 mm. Initially, the specimens were first worn for 30 cycles under a load of 300 N to remove the effect of surface slurry (pre-wearing), followed by cleaning and wearing for an additional 40 cycles (second wearing) to calculate the amount of mortar abrasion based on the following Equation (2):

(2) $$G=\frac{m_{1}-m_{2}}{0.0125}$$

where G is wear per unit area (kg/m^2^), $m_{1}$ is the mass of the specimen before wear (kg), $m_{2}$ is the mass of the specimen after wear (kg), and 0.0125 is wear area (m^2^). The average values of the results of the three specimens were taken as the wear results of the specimens.

#### 2.3.5. Microscopic Testing 

In order to specify the effect of SBR and SF on the microstructure of cement, Zeiss sigma300 scanning electron microscopy (Jena, Germany) was used to observe the cement specimen after 28 days of hydration (with a volume of less than 1 cm^3^). The cement cubes were ground, then soaked in absolute ethanol for 24 h, followed by drying at 60 °C for 12 h in preparation for XRD. Phase composition was tested using a D8 Advance X-ray diffractometer from Bruker, Germany, and measurements were made in the range of 10° to 80° at a rate of 10°/min.

#### 2.3.6. FT-IR 

Transmission infrared spectra of the samples were recorded using the Thermo Nicolet iS5 FT-IR spectrometer (Xi’an, China) over the wavenumber range of 4000 to 400 cm^−1^.

#### 2.3.7. BET 

The micropores and mesopores of the samples were tested using the BSDPS1 type fully automatic specific surface area and pore size analyzer produced by Best Instruments Technology Co., Ltd., Beijing, China.

## 3. Results and Discussion

### 3.1. Fluidity

**Figure 1:**
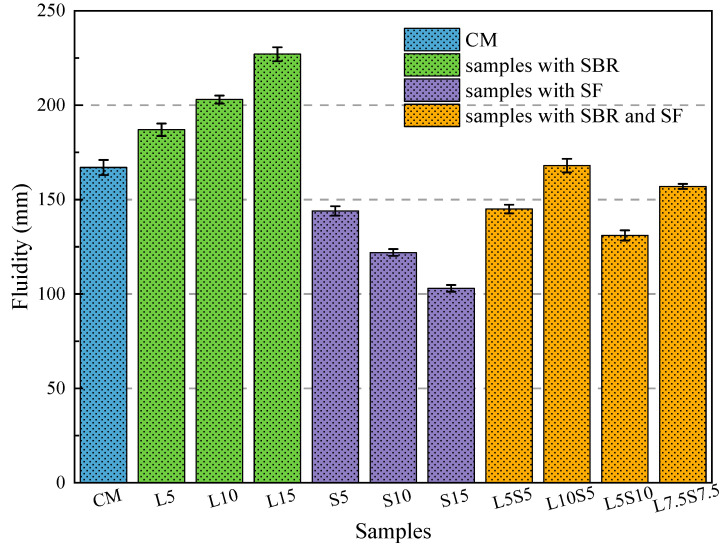
The influence of SBR latex and SF on the fluidity of cement mortar.

Figure 1 illustrates the impact of SBR latex and SF on the fluidity of cement mortar. The graph clearly demonstrates that SBR enhances the fluidity of mortar, with an increment from 167 mm to 227 mm at a relatively consistent rate as the substitution ratio increases. For each 5% increase in the latex substitution ratio, the mortar’s fluidity increases by 11.9%, 21.6%, and 35.9%. Researchers widely accept that SBR particles act as ball bearings, thereby improving workability [27]. Conversely, using SF as the substitute for cement produces the opposite effect. As the substitution rate of SF rises, the fluidity of the mortar decreases. The specimens S5, S10, and S15 experienced reductions in fluidity by 13.8%, 26.9%, and 38.3%. This is primarily attributed to the small particle size of SF, which increases the specific surface area and water demand as the substitution rate grows, consequently reducing the fluidity of the cement paste [28]. Furthermore, the fluidity of all compound-mixed specimens exceeded 140 mm, with L10S5 displaying a fluidity of 168 mm, nearly identical to the cement baseline specimen. After the cement was solely replaced, SF had a slightly greater impact on mortar fluidity compared to SBR latex. A lateral comparison reveals that with a total substitution rate of 10%, the fluidity of L5S5 decreased by 13.1% in comparison to CM. At the 15% total substitution rate, the fluidity of L7.5S7.5 decreased by 6% in comparison to CM. This suggests that for compound-mixed specimens, when the substitution rates of SBR latex and SF are equal, SF has a more pronounced effect on mortar fluidity. To offset the adverse impact of SF on fluidity, a higher latex substitution rate is required, as demonstrated by L10S5 and L5S10.

### 3.2. Drying Shrinkage

**Figure 2:**
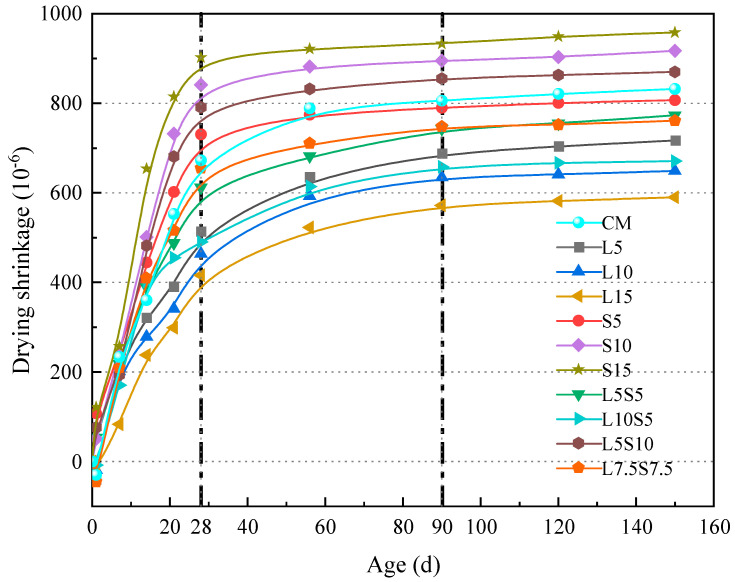
The effect of SBR latex and SF on the dry shrinkage of cement mortar.

Figure 2 illustrates the effects of SBR latex and SF on mortar drying shrinkage. It is evident that SBR latex significantly reduces the drying shrinkage of mortar. After 150 days, the shrinkage rates of L5, L10, and L15 decreased by 16.9%, 26.8%, and 35.5%. For SF replacement specimens, the shrinkage rate of S5 decreased by 3.7%, while S10 and S15 increased by 12.5% and 18.5%. This indicates that SF improves mortar drying shrinkage at low substitution rates, but higher rates lead to increased shrinkage due to the higher water absorption of SF. Regarding compound-mixed specimens, the shrinkage rate of L5S10 increased by 5.6%, whereas those of L5S5, L10S5, and L7.5S7.5 decreased by 8.7%, 23.6%, and 10.4%. The reduction in mortar drying shrinkage primarily results from reduced cement usage and the benefits of SBR latex and SF. As shown by the vertical dotted lines in the figure, based on the drying shrinkage trend, mortars can be categorized into two types, excluding the cement reference specimen CM. The first category, comprising S5, S10, S15, and L5S10, exhibits rapid shrinkage rate development within the initial 28 days, followed by stabilization until 150 days. The drying shrinkage development of this type of mortar is dominated by SF. Within the initial 28 days, SF rapidly reacts with Ca(OH)_2_, the cement hydration product, consuming a significant amount of water and increasing capillary pressure, thereby rapidly increasing the shrinkage rate. Beyond 28 days, as hydration nears completion, the shrinkage rate stabilizes. L5, L10, L15, L5S5, L10S5, and L7.5S7.5 fall into the second category, where the development of shrinkage is slower and generally lower. For these mortars, the shrinkage rate increases rapidly in the first 28 days, continues to grow significantly from 28 to 56 days, and then gradually stabilizes after 56 days. SBR latex is the primary influence on the drying shrinkage development in this mortar category. Both film-forming polymers and non-film-forming emulsion particles adhere to the surface of cement particles, delaying the hydration reaction, reducing the heat of hydration, and minimizing moisture evaporation. Additionally, the intertwined, interpenetrating elastic network formed by the polymer film and cement paste absorbs drying shrinkage stress, contributing to the reduced shrinkage rate of the mortar.

### 3.3. Mechanical Properties

#### 3.3.1. Compressive Strength

**Figure 3:**
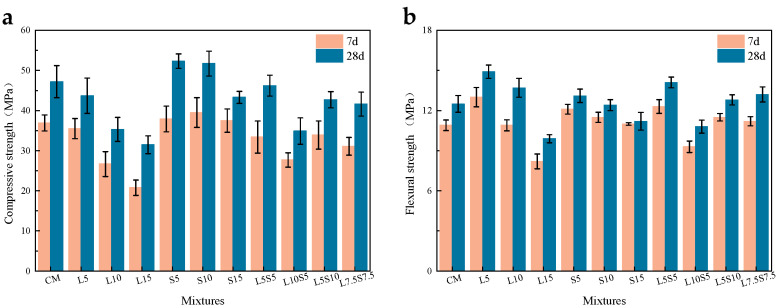
The influence of SBR latex and SF on the mechanical properties of cement mortar; (**a**) compressive strength; (**b**) flexural strength.

The influence of SBR latex and SF on the compressive strength of mortar is shown in Figure 3a. It can be seen from the graph that as the substitution rate of SBR latex increases, the 7-day and 28-day compressive strength of the mortar shows a continuous decreasing trend. Compared to the baseline specimen, the 28-day compressive strength of L5, L10, and L15 decreased by 7.4%, 25.2%, and 33.3%, with L10 and L15 being only 35.3 and 31.5 MPa. This indicates that when the substitution rate of SBR latex exceeds 10%, it causes significant damage to the compressive strength of the mortar. This is partly due to the reduction in cement usage and partly because the SBR latex adsorbs onto cement particles in the early stages, affecting hydration. Furthermore, the elastic modulus of the latex itself is much lower than that of cement, weakening the mortar’s load-bearing capacity under pressure, resulting in a decrease in compressive strength. Due to the reduction in cement usage, the enhancement of the early compressive strength of mortar by SF was not significant, with the 7-day compressive strength of S5, S10, and S15 being only slightly higher than the cement baseline specimen. It was found that, regardless of the amount of SF added, 3.92% to 5.4% of the SF participated in the pozzolanic reaction, with the remainder filling in the pores, explaining why the 28-day compressive strength of S5 and S10 were similar [29]. For the compound-mixed specimens, L5S5, L5S10, and L7.5S7.5 had lower early strength, but their 28-day compressive strengths were all above 40 MPa, with L5S5 having a compressive strength of 46.2 MPa, close to that of CM. This is because SBR latex not only impedes the hydration of cement particles but also delays the pozzolanic reaction of SF particles with hydration products [30]. The 7-day compressive strength of L10S5 was higher than L10 and L15, and the 28-day compressive strength was higher than L15 but lower than L10, which is because SF can enhance the early strength of mortar, albeit not enough to offset the negative impacts of reduced cement usage and SBR latex. Moreover, the 28-day compressive strength of L5S5 was 23.2% higher than L10 and 10.6% lower than S10; the 28-day compressive strength of L7.5S7.5 was 32.1% higher than L15 and only 3.9% lower than S15, which indicates that, at the same substitution rate, the strengthening effect of SF on mortar compressive strength can be stronger than the damage caused by SBR latex.

#### 3.3.2. Flexural Strength

The impact of SBR latex and SF on the flexural strength of mortar is illustrated in Figure 3b. It was observed that, with the increase in the substitution rate of SBR latex, the 7-day and 28-day flexural strength of the mortar initially increased and then decreased. The 28-day flexural strength of L5 and L10 increased by 19.2% and 9.6%, with the 7-day strength of L5 even surpassing the 28-day strength of CM. This occurred because SBR latex can form continuous polymer phases in the early stages, which gradually lose water and form a cross-linked network structure, significantly enhancing the later flexural strength of the mortar. However, the 28-day flexural strength of L15 was only 9.9 MPa, lower than the 7-day strength of CM. As the substitution rate increased, the flexural strength of S5 increased by 4.8%, while that of S10 only decreased by 0.1 MPa, and the strength of S15 decreased by 10%. This indicates that the impact of SF on the later-stage flexural strength of mortar was not significant, and even a substitution rate of 15% did not cause substantial damage to flexural strength. Moreover, it was observed that the 7-day flexural strength of S5, S10, and S15 all reached more than 90% of their 28-day flexural strength due to the primary role of SF in the early stages. In comparison to compressive strength, compound-mixed specimens showed greater improvement in flexural strength. The 7-day and 28-day flexural strength of L5S5, L5S10, and L7.5S7.5 were all higher than CM, and although the 28-day flexural strength of L10S5 was lower, it remained above 10 MPa. As the cement hydration process progressed, the SBR latex in the cement paste formed a film via dehydration. The polymer film and hydration products together formed an interwoven network structure, and SF improved the compactness of the mortar through secondary hydration and filling, ultimately resulting in a substantial increase in the flexural strength of the mortar, even with reduced cement usage.

### 3.4. Abrasion Resistance

**Figure 4:**
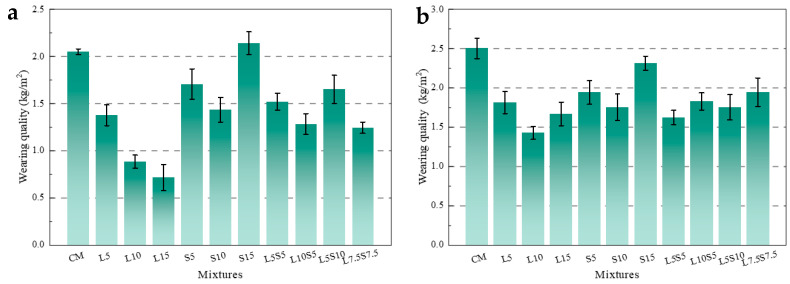
The influence of SBR latex and SF on the wear resistance of mortar; (**a**) pre-wearing; (**b**) second wearing.

**Figure 5:**
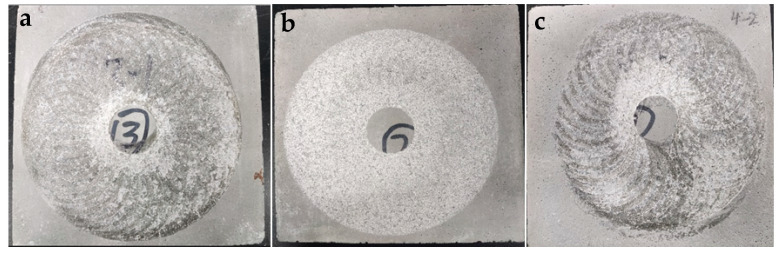
Surface topography of mortar after pre-wearing; (**a**) L10; (**b**) S10; (**c**) L10S5.

**Figure 6:**
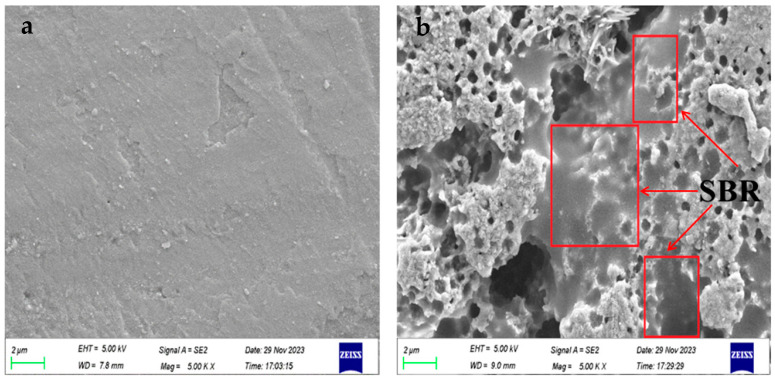
The microstructure of the mortar surface before and after pre-wearing; (**a**) before wear; (**b**) after wear.

The impact of SBR latex and SF on the abrasion resistance of mortar is depicted in Figure 4. Figure 4a reveals that after pre-wearing, the abrasion loss of L5 and L10 was reduced by 32.8%, 60%, and 65.1%. When the substitution rate of SBR latex reached 15%, the reduction in abrasion loss of the modified mortar was as high as 65.1%, but the decreasing trend tended to level off, indicating that the improvement effect of latex on the abrasion resistance of mortar gradually reached its limit. With the increase in the substitution rate of SF, the abrasion loss of mortar first decreased and then increased. The abrasion loss of S5 and S10 decreased by 16.7% and 30%, while the abrasion loss of S15 increased by 4.4%. Moreover, the compound-mixed specimens also performed well in terms of abrasion resistance. Figure 5a–c displays the surface morphology of L10, S10, and L10S5 mortar specimens after pre-wearing. It can be observed that there were grey–brown polymer films on the surfaces of L10 and L10S5, while S10 did not contain these. These polymer films were formed due to the SBR latex rising to the surface of the mortar during specimen formation. Figure 6a,b demonstrates the microstructural changes in the surface layer of the L10 mortar before and after pre-wearing. The surface of the mortar before wearing was primarily composed of cement products and was very smooth. From Figure 6b, it was observed that after abrasion, the mortar surface was filled with the network structure formed by SBR latex in between the cement stones, playing an important role in adhesion and cohesion. Coupled with the abrasion-resistant characteristics of the polymer itself, this ultimately led to improvement in the abrasion resistance of the mortar.

Figure 4b shows the abrasion loss of the mortar after the second wearing, which truly represents the abrasion resistance of the mortar. As shown in Figure 4, the abrasion loss of L5, L10, and L15 decreased by 27.5%, 43%, and 33.3%. This was due to the incorporation of SBR latex, where the polymer film formed continuous network structures within the cement stone, binding with the cement stone and sand to form the cohesive whole. The tensile stress of the polymer film fibers enhanced the flexibility and deformability of the cement mortar, effectively preventing wear of the mortar surface material. The abrasion loss of S5, S10, and S15 decreased by 22.4%, 30%, and 7.5%, indicating that although the cement usage was reduced, the active SiO_2_ in the SF could still react with Ca(OH)_2_ produced by cement hydration to form a large amount of calcium silicate hydrate (C-S-H)gel, promoting cement hydration and greatly improving the compactness of the cement mortar, thereby enhancing its abrasion resistance. Upon comparison, it was observed that, although the changing trends are similar and both achieved optimal results at the replacement rate of 10%, the enhancement effect of SBR latex on the abrasion resistance of mortar was significantly superior to that of SF. Additionally, the improvement in abrasion resistance for all four groups of blended specimens was above 20%, with L5S5 surpassing 35%, outperforming all single-blend specimens except for L10. This indicates that the combination of additives had a positive effect on enhancing the abrasion resistance of mortar, and the appropriate blending ratio was superior to the individual use of SBR latex or SF.

### 3.5. Composition and Pore Structure

#### 3.5.1. FT-IR and XRD

**Figure 7:**
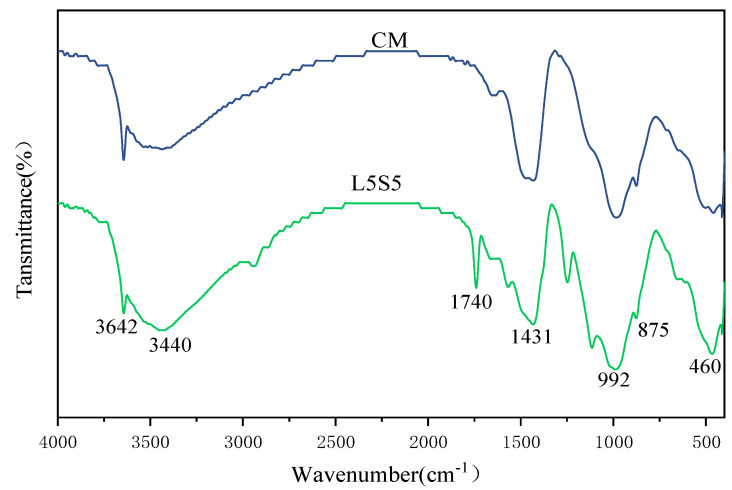
FT-IR diagram of CM and L5S5 pure pulp after 28 days of hydration.

The FT-IR spectrogram results of CM and L5S5 after 28 days of hydration are presented in Figure 7. The infrared absorption band at 460 cm^−1^ in the FT-IR spectrum corresponded to the symmetrical bending vibration of the Si-O-Si bond, indicating the presence of SiO_2_ in the product. The infrared absorption band at 870 cm^−1^ is the characteristic absorption band of ettringite (AFt). The transmittance of this absorption peak in CM was significantly higher than in L5S5, indicating a higher content of AFt in the mortar without SBR latex and SF. The infrared absorption band at 1431 cm^−1^ corresponded to the bending vibration of carbonate ions, indicating the presence of calcium carbonate in the mortar. The peak at 992 cm^−1^ corresponded to the -SiO_4_ phase and was present in both CM and L5S5 mortars. The infrared absorption band at 1740 cm^−1^ in the spectrum of L5S5 corresponded to C=O. Complexation between the carboxyl group of SBR latex and Ca^2+^ ensured that the polymer film adhered more closely to the hydration products, thus increasing the chemical adsorption between the hydration products and the polymer, which led to the formation of the polymer-hydration product-aggregate linkage structure, enhancing the cohesion of the composite material [31]. There was no C=O infrared absorption peak in the spectrum of CM. The absorption band at 3642 cm^−1^ was caused by the stretching vibration of the O-H bond and is characteristic of Ca(OH)_2_. This peak showed the same characteristics as the AFt absorption peak, indicating a higher content of Ca(OH)_2_ in the mortar without SBR latex and SF.

**Figure 8:**
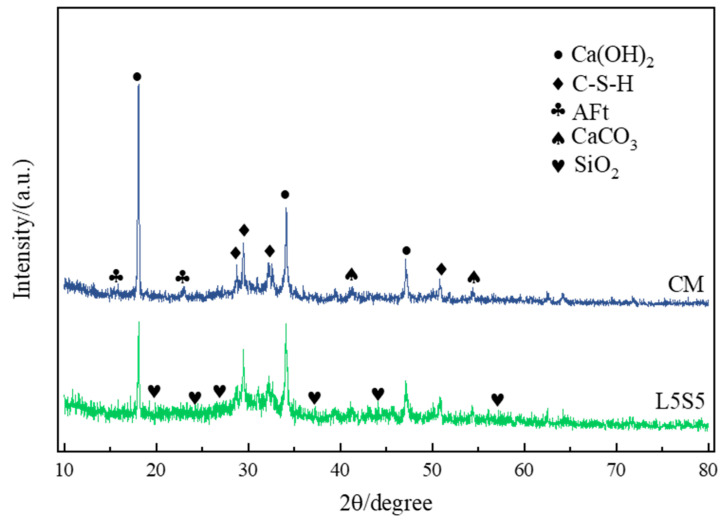
XRD diagram of CM and L5S5 pure pulp after 28 days of hydration.

Figure 8 shows the XRD pattern of CM and L5S5 pastes after 28 days of hydration. The main components of the pastes included Ca(OH)_2_, C-S-H, AFt, CaCO_3_, and SiO_2_, consistent with the phase composition results of the FT-IR test. The types of phase composition were basically the same in CM and L5S5, but the proportions of each product were significantly different. The Ca(OH)_2_ diffraction peak in CM was significantly higher than in L5S5, indicating a higher content of Ca(OH)_2_ in CM. The diffraction peaks of AFt and CaCO_3_ also showed the same characteristics as Ca(OH)_2_, suggesting higher contents of AFt and CaCO_3_ in CM. However, the C-S-H diffraction peak in L5S5 was higher than in CM, indicating the higher content of C-S-H in L5S5. On the one hand, the Ca^2+^ absorbed by the polymer film formed by SBR latex in L5S5 reduced the formation of Ca(OH)_2_ as the content of polymer in the mortar increased [32]. On the other hand, the active SiO_2_ in SF reacted with Ca(OH)_2_ in the secondary hydration reaction to form a large amount of C-S-H, thereby increasing its content. Additionally, the diffraction peak of SiO_2_ in L5S5 proved the presence of SF particles used for filling pores without participating in a pozzolanic reaction.

#### 3.5.2. BET

**Figure 9:**
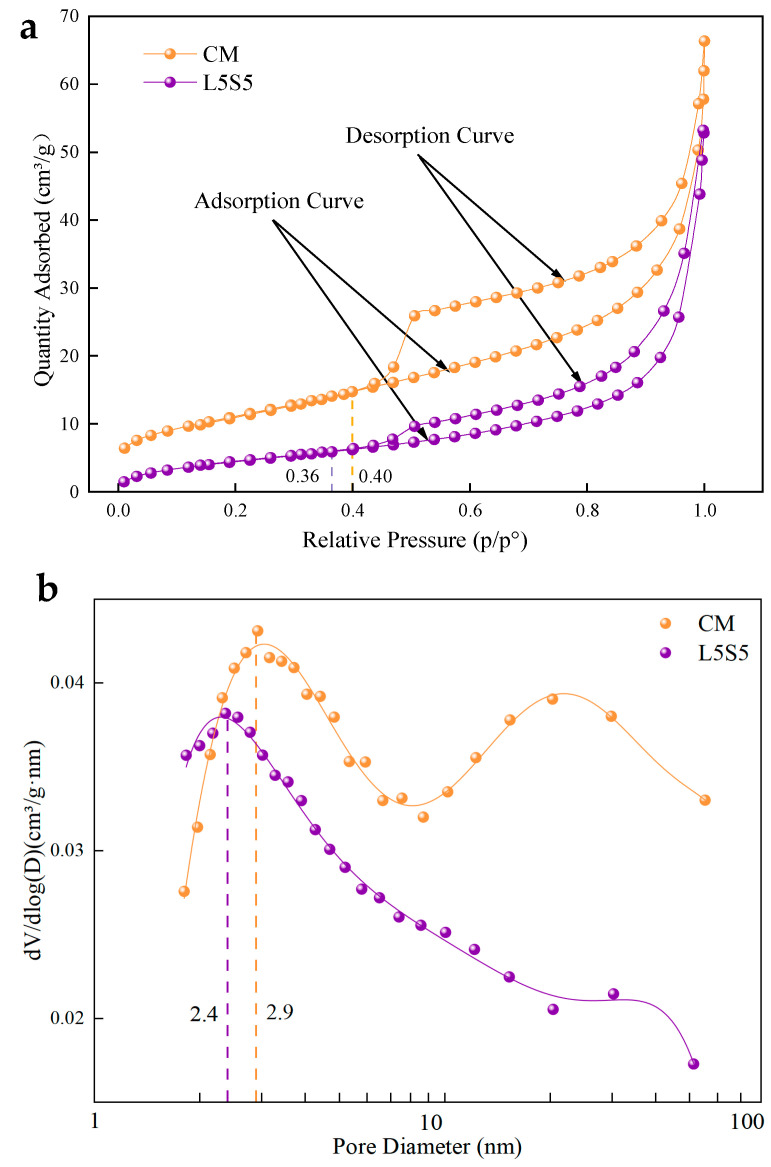
The pore structure of CM and L5S5 pure pulp after 28 days of hydration; (**a**) adsorption–desorption curve; (**b**) distribution of pore size.

Figure 9 shows the pore structure of CM and L5S5 pastes after 28 days of hydration as determined via BET. From Figure 9a, the separation point of the adsorption–desorption curve of CM corresponds to the relative pressure of 0.4, which is greater than the relative pressure of 0.36 at the separation point of the adsorption–desorption curve of L5S5. At the same time, the separation range of CM’s curve is larger and exhibits hysteresis, whereas L5S5’s curve has a smaller and smoother separation range. This indicates that under the combined action of SBR latex and SF, the pores in the cement paste were smaller, and the pore size distribution was more uniform. The pore size range of the cement paste determined via BET was 1~100 nm. From Figure 9b, based on the measured pore size distribution curve, the most probable pore size for CM within the 1~100 nm range was about 2.9 nm, and for L5S5, it was about 2.4 nm, with both representing gel pores. CM showed a broader peak around 20~40 nm, indicating a larger number of harmful pores, which were not present in L5S5. The BET test results revealed that in the range of 2~100 nm micro-pores, the cement paste with combined SBR latex and SF formed more and smaller micro-pores, which were transformed from larger pores. Therefore, the addition of SBR latex and SF, resulting in more hydration products and polymer film structures, refined the pore structure and optimized the pore size distribution, consistent with the FT-IR and XRD results.

## 4. Conclusions

In this study, cement was replaced with equal amounts of SBR latex and SF to observe the effects of both on the fluidity, drying shrinkage, mechanical properties, and abrasion resistance of cement mortar, and to investigate the mechanism of their effects. The main conclusions are as follows.

(1) The substitution of SBR latex can improve the flowability of mortar, while silica fume has the opposite effect. In the ternary system of cement–SBR latex–SF, SF has a more significant effect on the flowability of mortar. With the higher replacement rate of SF, the drying shrinkage of mortar is greater and more drastic, typically reaching a peak within 28 days and then stabilizing; SBR latex significantly reduces the drying shrinkage of mortar while also delaying its development.

(2) The combination of SBR latex and SF can reduce the adverse effect of latex on the compressive strength of mortar and increase its flexural strength; at a 10% dosage, improvement in the abrasion resistance of mortar by latex is far greater than that by SF. This is due to the dense polymer network in the cement greatly enhancing the abrasion resistance of the mortar.

(3) The combined use of SBR latex and SF can effectively improve the microstructure of mortar. The incorporation of SBR latex and SF refines the pore size of the mortar due to the polymer structure formed by the latex and the secondary hydration products of SF, inhibiting the formation of harmful pores, enhancing the compactness of the mortar, and consequently greatly improving the macroscopic performance of the mortar for pavement.

## Figures and Tables

**Table 1:** Chemical composition of cement and silica fume (wt%).

Materials	SiO_2_	Al_2_O_3_	Fe_2_O_3_	CaO	MgO	SO_3_	Na_2_O_eq_	LOSS	f-CaO
cement	22.9	7.2	2.6	57.8	2.6	2.5	0.3	3.5	0.6
silica fume	98	0.7	0.5	0.2	0.6	-	-	-	-

**Table 2:** Concrete proportion and performance.

Sample	w/b	Water (g)	Cement (g)	SBR Latex (g)	SF (g)	Quartz Sand (g)	Defoamer (g)
CM	0.40	240	600	0	0	1200	3.6
L5	0.40	210	570	60	0	1200	3.6
L10	0.40	180	540	120	0	1200	3.6
L15	0.40	150	510	180	0	1200	3.6
S5	0.40	240	570	0	30	1200	3.6
S10	0.40	240	540	0	60	1200	3.6
S15	0.40	240	510	0	90	1200	3.6
L5S5	0.40	210	540	60	30	1200	3.6
L10S5	0.40	180	510	120	30	1200	3.6
L5S10	0.40	210	510	60	60	1200	3.6
L7.5S7.5	0.40	195	510	90	45	1200	3.6

## Data Availability

The data used to support the findings of this study are included within the article.
